# Optimization of Light Intensity and NaNO_3_ Concentration in Amazon Cyanobacteria Cultivation to Produce Biodiesel

**DOI:** 10.3390/molecules24122326

**Published:** 2019-06-24

**Authors:** Joseline Barbosa Aboim, Deborah Terra de Oliveira, Vanessa Albuquerque de Mescouto, André Silva dos Reis, Geraldo Narciso da Rocha Filho, Agenor Valadares Santos, Luciana Pereira Xavier, Alberdan Silva Santos, Evonnildo Costa Gonçalves, Luis Adriano Santos do Nascimento

**Affiliations:** 1 Laboratory of Oils of the Amazon, Guamá Science and Technology Park, Federal University of Pará, Belém, Pará 66075-750, Brazil; joseline.aboim@gmail.com (J.B.A.); deborahterra.o@gmail.com (D.T.d.O.); vanessadmescouto@gmail.com (V.A.d.M.); narciso@ufpa.br (G.N.d.R.F.); 2 Graduate Program in Biotechnology, Federal University of Pará, Belém, Pará 66075-110, Brazil; agenorvaladares@yahoo.com.br (A.V.S.); luxavier@gmail.com (L.P.X.); alberdan.ufpa@gmail.com (A.S.S.); evogoncalves@gmail.com (E.C.G.); 3 Graduate Program in Chemistry, Federal University of Pará, Belém, Pará 66075-110, Brazil; andrechemistry25@gmail.com

**Keywords:** cyanobacteria, lipids, cultivation optimization, biodiesel quality parameters

## Abstract

The objective of this study, for the first time, was to optimize Amazonian cyanobacterial culture conditions for improving cell productivity and lipid content, by analyzing the effect of light intensity and nitrogen concentration, for empirically evaluating biodiesel quality parameters. The strains *Synechocystis* sp. CACIAM05, *Microcystis aeruginosa* CACIAM08, *Pantanalinema rosaneae* CACIAM18, and *Limnothrix* sp. CACIAM25, were previously identified by morphological and molecular analysis (16S rRNA) and were selected based on their production of chlorophyll a and dry cell weight. Then, factorial planning (2^2^) with central points was applied, with light intensity and NaNO_3_ concentration as independent variables. As response variables, cell productivity and lipid content were determined. Statistical analysis indicated that for all strains, the independent variables were statistically significant for cell productivity. Analysis of the fatty acid composition demonstrated diversity in the composition of the fatty acid profile from the experimental planning assays of each strain. The Biodiesel Analyzer software predicted the biodiesel quality parameters. CACIAM05 and CACIAM25 obtained better parameters with low levels of light intensity and NaNO_3_ concentration, whereas CACIAM08 and CACIAM18 obtained better parameters with low NaNO_3_ concentrations and high luminous intensity.

## 1. Introduction

Cyanobacteria are promising sources of fuel, where they can be used as a source of raw material for generating energy, such as for producing hydrogen, lipids for biodiesel due to the high lipid content and productivity, hydrocarbons for gasoline, and carbohydrates for ethanol. Another important focus in the energy industry is the conversion of ethanol from carbon sequestration, besides the accumulation of glycogen, lipids, and hydrocarbons to other types of biofuels. Such microorganisms are renewable and can lead to a reduction in CO_2_ emission through mechanisms that remove CO_2_ from the air and convert the C to lipids, carbohydrates, and proteins [1,2,3,4]. Another advantage in the use of cyanobacteria is their ability to grow in various locations, including those considered unfit for agricultural development or in wastewater streams, which makes it possible to explore new areas for biodiesel production [5]. 

Lipid production in thylakoid membranes is associated with high rates of rapid growth and photosynthesis, which is ideal for biodiesel production [6,7,8]. Biodiesel is the most sustainable and renewable alternative to diesel and is defined as a blend of fatty acid alkyl esters. It is produced by a transesterification reaction, in which triacylglycerides react with short-chain alcohols to form alkyl esters [9]. Considering that the properties of the fuels are partly dependent on the composition of fatty acids, the determination of the profile of the fatty acid constituent of biomass is of extreme importance for the quality of the biodiesel that will be synthesized [10,11].

Under normal conditions, cyanobacteria are generally cultured in vitro in a BG-11 medium at 30 °C under agitation and illumination of 50 µmol photons m^−2^ s^−1^ [12], the culture being the main form of biomass obtainment [7]. The production of oils from microalgae and cyanobacteria biomass is related to the cultivation conditions to which they are subjected and can stimulate or suppress lipid productivity [13,14]. The optimization of components of crop media, such as CO_2_, H_2_O, N_2_, and factors such as light intensity, pH, and temperature influence the growth of biomass and the lipid content [7]. Studies have been directed to cultivation conditions to increase lipid and biomass production and thus to adjust the profile of fatty acids, affording a better-quality biodiesel [15,16].

The objective of this study was to optimize, for the first time, Amazonian cyanobacterial culture conditions to improve the biomass and lipid content and analyze the effect of light intensity and nitrogen concentration with samples used in previous studies [17]. Although there is no consensus in the literature on the influence of light intensity on the lipid composition, especially on the fatty acid profile [18,19], as the cyanobacterial strains were collected in the Amazon, which is known for a high incidence of solar rays practically all throughout the year, it was interesting to investigate if there was adaptation in these strains, with respect to these light rays in the variables studied in the present work. In addition, the NaNO_3_ concentration is known in the literature as an important altering factor when optimized, where it increases the lipid percentage and biomass yield [20,21,22,23].

Statistical analysis through the ANOVA method (Supplementary Materials), with results being presented in Pareto graphs and surface graphs, showed if the independent variables, luminous intensity, and NaNO_3_ concentration and their integration were statistically significant in the production of biomass and lipid content, as well as whether the independent variable had positive or negative effects on each response variable, namely biomass productivity and lipid content.

In addition, another objective of this study was to investigate the potential of cyanobacteria in the Amazon region, which is known for rich biodiversity, to show the usefulness of working with local strains, since there would be no disturbance to local biodiversity when using imported allochthonous strains. The study deals with the continuation of a pioneering line of research in the Amazon, using strains collected in the region as a way of demonstrating the potential use of biofuel production, and for other purposes, such as cosmetic uses [17,24]. The hypothesis of the present study is that by combining high light intensities with limitation of the nitrogen source, the production of organic compounds, such as lipids, is increased. The hypothesis considers that the cyanobacteria were collected in an environment with high solar radiation, so they are already genetically adapted to improve their organic production in these conditions, following previous studies in the literature, where nitrogen limitation leads to an increase in lipid production.

## 2. Results and Discussion

### 2.1. Selection of Strains by Determination of Chlorophyll a

**Figure 1:**
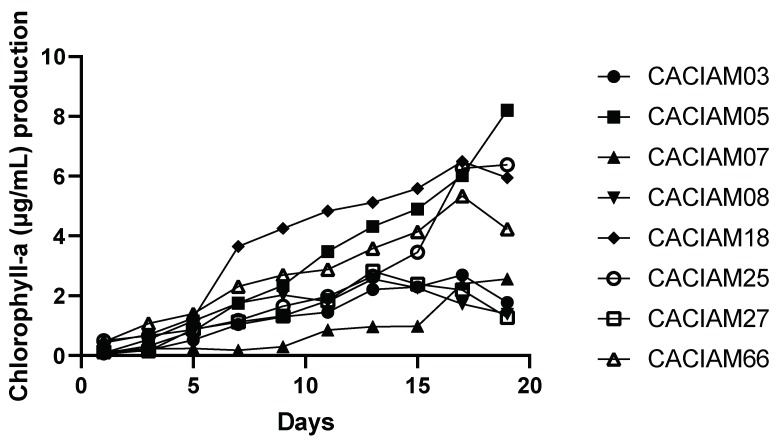
Growth curve by extraction of chlorophyll a from cyanobacteria strains. Data are mean values of three replicate cultures.

The monitoring of the growth of the cyanobacteria by chlorophyll a determination allowed the identification of lines that present higher biomass production, without the optimization of culture conditions (Figure 1).

The strains that presented the highest concentration of chlorophyll a were *Synechocystis* sp. CACIAM05, *Limnothrix* sp. CACIAM25, *Pantanalinema rosaneae* CACIAM18, and *Synechococcus* sp. CACIAM66. The strains chosen to follow the culture optimization study were the filamentous *Limnothrix* sp. CACIAM25, *Pantanalinema rosaneae* CACIAM18, the unicellular *Synechocystis* sp. CACIAM05, and *Microcystis aeruginosa* CACIAM08. The choice of the *Microcystis aeruginosa* CACIAM08 is justified by the species already having been widely studied, and the biomass quality is recognized for biofuel production, hence the interest in studying it, despite the low productivity of biomass [25,26,27,28,29].

### 2.2. Influence of Light Intensity and NaNO_3_ Concentration on Cyanobacteria Cultivation

As a way of optimizing the culture conditions, CACIAM05, CACIAM08, CACIAM18, and CACIAM25 were selected based on their chlorophyll a (μg/mL) production. A factorial design (2^2^) was applied with central points, with light intensity (X) and the concentration of NaNO_3_ (Y). As response variables, the lipid content (%) and the biomass productivity determined at the end of 13 days of cultivation were selected. The values obtained and the experimental matrix are both presented in Table 1.

#### 2.2.1. Statistical Analysis of the Factorial Planning of Strain Cultivation with Biomass Productivity as the Response Variable

Table 1 shows the biomass yields for the strains after 20 days of cultivation, where CACIAM05 ranged from 26.8 to 48.4 (mg/L/day), CACIAM08 ranged from 9.2 to 18.4 (mg/L/day), CACIAM18 ranged from 23.1 to 45.8 (mg/L/day), and CACIAM25 ranged from 24.1 to 53.7 (mg/L/day).

In all the strains, the highest values of biomass yield were at the higher levels of light intensity and NaNO_3_ concentration (Assay 4). In the same way, Zulkifli et al. [20] found that the nutrient composition, especially nitrogen, affects both cell growth and the biochemical composition of microalgae. Similarly, among the factorial design trials, except for CACIAM08 at the central point, the lowest biomass yields found for all strains were at the low levels of both light intensity and NaNO_3_ concentration (Assay 1). 

**Figure 2:**
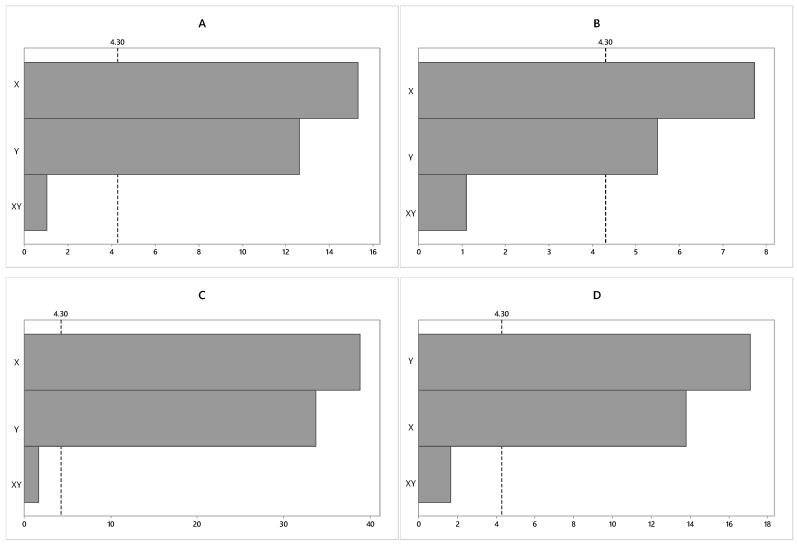
Effect of the independent variables (X: Light intensity and Y: NaNO_3_ concentration) and their interaction (XY) on biomass productivity (mg/L/day) for a 95% confidence level. (**A**) *Synechocystis* sp. CACIAM05; (**B**) *Microcystis aeruginosa* CACIAM08; (**C**) *Pantanalinema rosaneae* CACIAM18; (**D**) *Limnothrix* sp. CACIAM25.

In Figure 2, the statistical analysis of the experimental design for the biomass productivity response variable (mg/L/day), obtained from the CACIAM05, is shown in the Pareto charts. In the Pareto graph, the bars are the standardized (*t*-calculated) effects related to biomass productivity. Therefore, variables that exceed the dashed vertical line (*t*-table) are considered statistically significant at a 95% confidence level.

From the Pareto graph (Figure 2), it can be inferred that, for all strains, both light intensity and nitrogen concentration (independent variables) were significant at the 95% confidence level, presenting positive values on the productivity of biomass (the response variable). It was observed that the light intensity was the variable that most influenced biomass productivity, with a positive effect on the biomass productivity (mg/L/day). Regarding the NaNO_3_ concentration, it also showed a positive effect (12.60) on the biomass productivity (mg/L/day). This result indicates that the increase in light intensity and the concentration of NaNO_3_ in the culture medium contributes to an increase in the biomass productivity (mg/L/day) in the studied strains. The interaction between variables was not statistically significant at the 95% confidence level.

In thylakoid membranes, reactions occur that convert light energy into chemical energy. Photosynthesis reactions are divided into light and dark phases. In the light reaction centers, there is a release of photons to oxidize water and a reduction of NADP in NADPH. The products of this reaction are O_2_ and ATP. The energy obtained in this phase is used to produce organic compounds, such as lipids. The chemical energy, stored in the form of NADPH and ATP (reaction products accumulated by light phase photosynthesis), is used to power light-independent reactions to produce organic compounds as lipids [30]. For this reason, many studies improve light intensity to increase cyanobacteria and microalgae lipid production, and other products that are advantageous to producing biodiesel [31,32,33,34].

**Figure 3:**
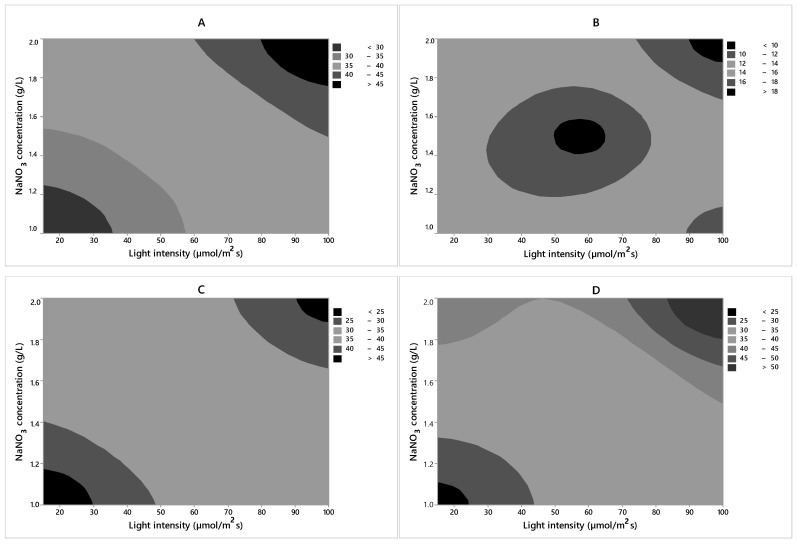
Level curves for the biomass productivity (mg/L/day) as a function of the independent variables for each strain. (**A**) *Synechocystis* sp. CACIAM05; (**B**) *Microcystis aeruginosa* CACIAM08; (**C**) *Pantanalinema rosaneae* CACIAM18; (**D**) *Limnothrix* sp. CACIAM25.

From the contour plot (Figure 3) provided by the Minitab 7.0 program, it was possible to investigate the optimum biomass productivity region for strains among the values of the independent variables studied in the experimental design. Peng et al. [35] found the cell growth improved significantly when the sodium nitrate concentration increased. In the same study, it was found that an excess of nitrogen inhibits growth, which suggests that lipids accumulate when the nitrogen concentration is low, rather than when it is in excess. However, biomass productivity was reduced. The experimental design was based on the high level of light intensity (100 µmol photons m^−2^ s^−1^) and high NaNO_3_ concentration (2 g/L). In the suggested region, a biomass productivity value above 45 mg/L/day can be obtained for CACIAM05, 18 mg/L/day for CACIAM08, 45 mg/L/day for CACIAM18, and 50 mg/L/day for CACIAM25.

Nitrogen is generally considered as one of the main nutrients for cyanobacteria growth in temperate and subtropical areas, because this element is mainly used to form proteins and nucleic acids. Many studies have shown that nitrogen limitation can decrease growth and increase polysaccharide production in algae. The synthesis of glycosides or polysaccharide by microalgae during cultivation can cause autoflocculation, which improves the biomass recovery efficiency without the use of chemical reagents [31,32,33]. 

#### 2.2.2. Statistical Analysis of the Factorial Planning of Strain Cultivation with Lipid Content as the Response Variable

It can be inferred from Table 1 that the lipid content (%) for CACIAM05 ranged from 15.3 to 25.6(%), CACIAM08 ranged from 12.37 to 43.97(%), CACIAM18 ranged from 20.6 to 37.9(%), and CACIAM25 ranged from 7.0 to 58.3(%).

Except for the CACIAM08, the highest values of lipid content were obtained with a high level of light intensity and a low concentration of NaNO_3_ (Assay 3). For CACIAM08, the highest value obtained for the lipid content was at high levels of both light intensity and NaNO_3_ concentration. In contrast, the lowest lipid content values for CACIAM05 and CACIAM08 were found at low levels of both light intensity and NaNO_3_ concentration (Assay 1). For the strains CACIAM25 and CACIAM18, the lowest value of lipid content was found at medium levels of light intensity and NaNO_3_ concentration (Assay 7). The strain with the highest value of lipid content was CACIAM25.

**Figure 4:**
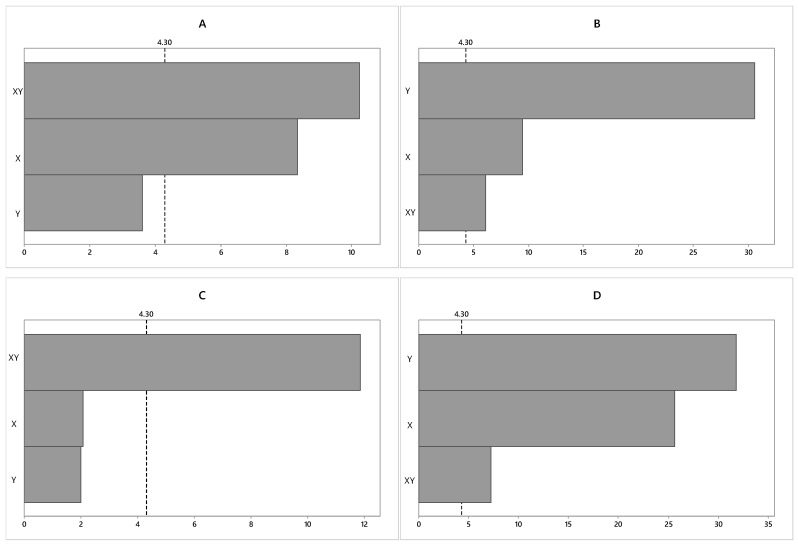
Effect of the independent variables (X: Light intensity and Y: Nitrogen concentration) and their interaction (XY) on lipid content (%), for a confidence level of 95%. (**A**) *Synechocystis* sp. CACIAM05; (**B**) *Microcystis aeruginosa* CACIAM08; (**C**) *Pantanalinema rosaneae* CACIAM18; (**D**) *Limnothrix* sp. CACIAM25.

Figure 4 presents the Pareto and interaction graphs of the experimental design statistical analysis for the response variable lipid content (%), as obtained from the studied strains. Variables that exceed the dashed vertical line (*t*-table) are considered statistically significant at the 95% confidence level. 

In the case of the CACIAM05, both the luminous intensity and the interaction between the two independent variables were statistically significant at a 95% confidence level. The interaction between the variables was the factor that presented the main negative effect (−10.25), followed by the luminous intensity, which was indicated to have a positive effect (+8.34) on the lipid content (%). Regarding the NaNO_3_ concentration, it was not statistically significant at the same confidence level, suggesting that nitrogen levels have no influence on the biomass productivity of the strain. This result indicates that in order to obtain a higher content of lipids (%) at the end of culturing, it is necessary to increase the luminous intensity and decrease the interaction between the light intensity and NaNO_3_ concentration.

For the strains CACIAM08 and CACIAM25, all the variables were significant at a 95% confidence level. For the strain CACIAM08, the increase in luminous intensity and the concentration of NaNO_3_ in the culture medium contributed to the increase in lipid content. For CACIAM25, the NaNO_3_ concentration was responsible for the main negative effect (−31.79), followed by the positive effect of light intensity (+25.63) and the negative effect of the interaction between the variables (−7, 26). This result indicates that, within the cultures, low amounts of nitrates produce high amounts of lipids, while high amounts of light increase these levels even further.

For CACIAM18, only the interaction between the variables was significant, with a negative effect (−11.84), whereas the light intensity and NaNO_3_ concentrations analyzed separately were not significant at a 95% confidence level. 

**Figure 5:**
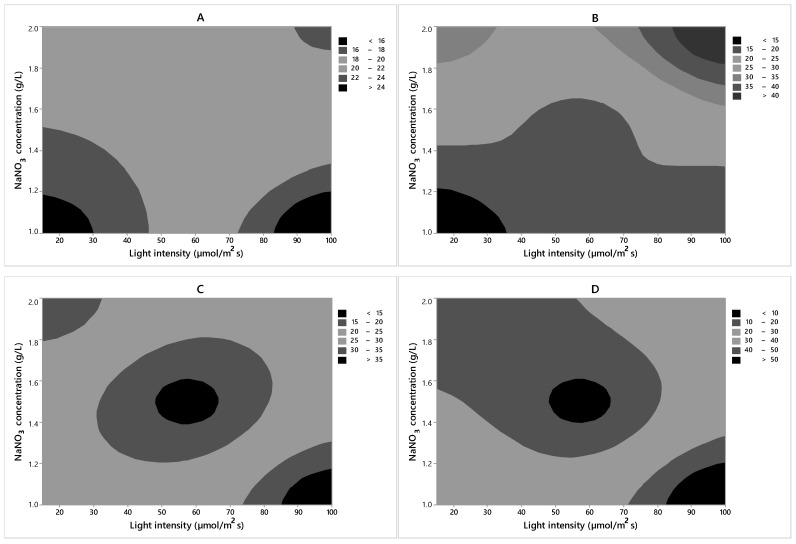
Level curves for the lipid content (%) as a function of the independent variables for each strain. (**A**) *Synechocystis* sp. CACIAM05; (**B**) *Microcystis aeruginosa* CACIAM08; (**C**) *Pantanalinema rosaneae* CACIAM18; (**D**) *Limnothrix* sp. CACIAM25.

From the contour plot (Figure 5) provided by the Minitab 7.0 program, it was possible to investigate the optimum region for increasing the lipid content for the evaluated strains, based on the values of the independent variables studied in the planning experiment. Accordingly, for CACIAM05, CACIAM18, and CACIAM25, the experimental design should be carried out with a high light intensity (100 µmol photons m^−2^ s^−1^) and low NaNO_3_ concentration (1 g/L). For the strain CACIAM08, the experimental design should be carried out with a high light intensity (100 µmol photons m^−2^ s^−1^) and high NaNO_3_ concentration (2 g/L).

Following a similar study, Cuellar-Bermudez et al. [36] investigated the change in light intensity (0–1920 µmol photons m^−2^ s^−1^), CO_2_ concentration (1 to 5% *v/v* of total air) and pH (6–11) in the cultivation of *Synechocystis* sp. PCC6803. In general, the total lipid content increased proportionally with light intensity and was higher at pH 9 (3% CO_2_).

The effect of light intensity (50–150 µmol photons m^−2^ s^−1^) and the concentration of Na_2_ CO_3_ (0.5–1.5 g) was studied by Da Rós et al. [7], who optimized the culture conditions of *Microcystis aeruginosa* NPCD-1 according to a complete factorial design of 2^2^. The highest values of biomass productivity were found at the central point of both variables (luminous intensity equivalent to 100 µmol photons m^−2^ s^−1^ and Na_2_CO_3_ concentration equal to 1.0 g/L). In contrast, the highest lipid content was found at the high points of the variables (luminous intensity equivalent to 150 µmol photons m^−2^ s^−1^ and Na_2_CO_3_ concentration equal to 2.0 g/L).

### 2.3. Empirical Parameters of the Biodiesel Quality from the Fatty Acid Profile

Using empirical equations based on the fatty acid profile (Table 2), biodiesel properties can be predicted by Biodiesel Analyzer, calculating the degree of unsaturation (DU), saponification value (SV), iodine value (IV), cetane number (CN), long chain saturated factor (LCSF), cold filter plugging point (CFPP), cloud point (CP), pour point (PP), allylic position equivalent (APE), bis-allylic position equivalent (BAPE), higher heating value (HHV), kinematic viscosity (ν), and density (ρ) (Table 3).

It can be observed that, in general, the profile detected of fatty acids of the species when grown at different levels of luminous intensity and NaNO_3_ concentration is different. Except for the CACIAM08 assay at a low NaNO_3_ concentration and high light intensity, all other assays presented a 16:0 acid in their composition. A high level of fatty acid (16:0) was observed, which is commonly found in bacteria and cyanobacteria [19].

Another fatty acid abundant among the studied strains, except for strain CACIAM18, was C18:1, which ranged from 4.59 to 49.50%. Similarly, the C17:0 fatty acid was quantitative, except for CACIAM18, which ranged from 2.06 to 29.45%. Seyfabadi et al. [18] found that the production of long-chain saturated fatty acids may help in dissipating excess light energy and preventing photochemical damage to algal cells. To corroborate, Renaud et al. [37] found increases in the production of saturated fatty acids at higher light intensities, indicating interruptions of the biosynthetic processes of chain elongation and desaturation.

Economou et al. [38] explain that the absence of long chain aliphatic fatty acids, as well as the high amount of saturated and monounsaturated fatty acids (almost 80%) in cyanobacteria are parameters that indicate promising raw material for biodiesel production. The fatty acid present in all assays was C18:1, in which, from the central point, there was a 66% increase in the high levels of both variables, namely 47% in the high level of NaNO_3_ concentration and in the low level of light intensity, a 81% reduction in the low level of NaNO_3_ concentration and the level of light intensity, and 48% at low levels of both variables. 

Zili et al. [19] reported that the C16:0 and C18:0 proportion increased at NaNO_3_ high concentration. For unsaturated fatty acids, it was observed they increased at high light intensity and UV radiation, suggesting that PUFAs have an effect protector against extreme light conditions. Colla et al. [39], when evaluating variations in temperature and nitrogen concentration, found significant changes in the synthesis of palmitoleic (C16:1) and linoleic (C18:2) acids. Specifically, at a temperature of 35 °C, an increase in the concentration of the aforementioned acids was observed.

The results obtained in the present study were compared with standards of the Brazilian National Petroleum Agency (ANP), the American Society of Testing and Materials (ASTM) and the European Standard UNE-EN 14214 (Table 3).

For all strains, it was observed that the cetane number was in accordance with the European and American standards and the iodine number was in accordance with the European standard. Only CACIAM25 at a high NaNO_3_ concentration and low light intensity was within the European standard for density.

Except for the CACIAM08 assay at the low NaNO_3_ concentration and high light intensity, the values found for kinematic viscosity for the strains are in accordance with the United States (US) standards. Only the CACIAM08 assays at the high levels of NaNO_3_ concentration and light intensity, CACIAM08 at the high NaNO_3_ concentration and low light intensity, CACIAM18 at the low levels of both variables, and all trials of CACIAM25 agree with both the Brazilian and European norms.

Note that the best values obtained for the biodiesel quality parameters were found in the CACIAM08 assay at a low NaNO_3_ concentration and high luminous intensity, CACIAM05 at low levels of both variables, CACIAM18 at a low NaNO_3_ concentration and high luminous intensity, and CACIAM25 at low levels of both variables.

Aboim et al. [17] cultivated CACIAM18 and CACIAM25 in ASM-1 and BG-11 culture media, extracted the lipids, esterified the fatty acids, and obtained the methyl esters, which were subjected to a chromatography step that identified the fatty acid profile. For the BG-11 culture medium used in the present study, the factorial design results of the present study for the biodiesel quality parameters calculated empirically based on the fatty acid profile were within what has been found in the literature.

The degree of unsaturation refers to the fatty acid profile. When compared to diesel, fatty acid unsaturation leads to greater biodiesel instability, considering that the oxidation process starts primarily at allylic positions on the carbon atoms neighboring the double bond [40]. Oxidation stability is one of the main issues affecting the use of biodiesel, due to its content of polyunsaturated methyl esters [41]. Therefore, the strains with the highest oxidative stability, based on the data of the degree of establishment, equivalent allyl position, bis-allylic equivalent, and oxidative stability (data not shown) for each strain were the following: CACIAM05 at high levels of both independent variables; CACIAM08 and CACIAM18 at low NaNO_3_ concentration and high luminous intensity; and CACIAM25 at low levels of both variables

For oleaginous microalgae, the IV has values that range from 111–135 g iodine/100 g of oil, and the SV has values that range from 205–208 mg KOH g^−1^ of oil. These values are considered high [42]. Martínez et al. [43] presented a SV based on the prediction of the fatty acid profile and the biodiesel. While the parameters were determined empirically, the values 193.2 and 184.0 mg KOH/g for soybean and sunflower, respectively, were found. When the values were determined from the biodiesel, values of 190.7 and 185.0 mg KOH/g were obtained for the soybean and sunflower, respectively. This result suggests that the parameters presented in Table 3 should follow the same pattern after the transesterification reaction and the consequent biodiesel production. 

The LCSF was calculated to derive the CFPP, which is impaired by lipid raw materials with long chains of saturated fatty acids. It has been described in the literature that the quality of biodiesel is higher when it possesses high levels of monounsaturated fatty acids (mainly oleic acid) [7,44]. The values obtained for the species studied varied from −9.13 to 21.19 °C, which indicated that crystallization would not occur in countries with tropical climates, except for CACIAM08, which was cultured at high levels of both independent variables.

The cloud point and flow point indicate the lowest temperatures at which crystal formation is observed and to which the lubricating oil can flow, respectively. Only CACIAM05, both at high and low levels of both variables, presented values above the ambient temperature of tropical climates, which may cause a decrease in engine performance when the oil is used.

The parameters of heating value, viscosity, and density all increased as the length of the chain (number of carbon atoms) increased, presenting the highest values in the tests that showed saturated fatty acids in a greater proportion. Thus, it can be observed that the amount of monounsaturated fatty acids is directly proportional to the described parameters.

## 3. Materials and Methods 

### 3.1. Obtaining the Strains and Cultivation

The strains *Microcystis aeruginosa* CACIAM03, *Synechocystis* sp. CACIAM05, *Lyngbya* sp. CACIAM07, *Microcystis aeruginosa* CACIAM08, *Synechococcus* sp. CACIAM66, *Pantanalinema rosaneae* CACIAM18, *Limnothrix* sp. CACIAM25, and *Planktothrix pseudoagardhii* CACIAM27 used in this study were provided by the Laboratory of Biomolecular Technology (Institute of Biological Sciences/UFPA, Belém, Pará, Brazil). The strains were collected at the hydroelectric plant of Tucuruí Lake (CACIAM03, CACIAM07, CACIAM08, CACIAM25, CACIAM27, and CACIAM66) and the Bologna reservoir (CACIAM05 and CACIAM18). All strains were previously identified using morphological analysis and some strains were identified by molecular (16S rRNA) analysis. The GenBank accession numbers for the 16S rRNA of CACIAM03, CACIAM05, and CACIAM66 are, respectively, MG272376.1, MG272377.1, and MG272380.1. For the other strains, BLAST identification, based on the 16S rRNA sequence, returned the following identities: CACIAM07 (98.82% with *Lyngbia* sp. (KP178669.1)), CACIAM08 (99.5% with *Microcystis aeruginosa* (MK589720.1)), CACIAM18 (90.44% with *Pantanalinema rosaneae* (KY873318.1)), CACIAM25 (99.49% with *Limnothrix* sp. (LC272581.1)) and CACIAM27 (96.63% with *Planktothrix pseudagardhii* (FJ184390.1)).

The strains were cultured in ASM-1 (CACIAM08 and CACIAM27) and BG-11 (CACIAM03, CACIAM05, CACIAM07, CACIAM66, CACIAM18, and CACIAM25) by methods reported by Gorham et al. [45] and Allen [44]. From a 19–28 days stock culture, 3 mL was taken and inoculated in a 120 mL fresh ASM-1 or BG-11 medium. The choice of culture media was made based on a previous study that identified the ideal culture medium for each strain [17]. The culture time was 20 days. The strains were incubated in a biochemical oxygen demand (BOD) in triplicate (NovaInstruments NI 1717) (Novainstruments Equipamentos para Laboratórios Ltda, Piracicaba, São Paulo, Brazil; −110 V) type incubator with a photoperiod of 13 h light and 11 h dark at 23 ± 2 °C, and the luminous intensity measured with a luxmeter (Minipa model MLM-1011) (Minipa do Brasil Ltda, Joinville, Santa Catarina, Brazil) was 57.5 µmol photons m^−2^ s^−1^. The strains in this step were cultured under the same conditions of time, temperature, and illumination.

In the second stage of the work, the strains chosen to continue the cultivation study (CACIAM05, CACIAM08, CACIAM18, and CACIAM25) were cultivated. From a 20 days stock culture, 15 mL was taken and inoculated in a 600 mL fresh ASM-1 or BG-11 medium for 13 days, following the same conditions mentioned above to obtain the biomass productivity and lipid content.

### 3.2. Measurement of Growth

#### 3.2.1. Biomass Productivity (Determination of Chlorophyll a)

This experiment was done to select four strains, among the eight initially studied, which have a higher productivity of biomass by the method of chlorophyll a, to follow the optimization of culture conditions, to improve those that already have high growth rates. The quantification procedure was performed per Meeks and Castenholz [46] with modifications suggested by Fiore et al. [47] every 2 days for 20 days. Three milliliters were collected from each culture and centrifuged for 4 min at 4000× *g*, after which the supernatant was discarded and the pellet was collected. Subsequently, 3 mL of a 9:1 methanol and water mixture was added to the pellet, homogenized, and left for 15 min in the dark for maximum chlorophyll extraction, then, the contents were centrifuged for 15 min at 4000× *g*. The absorbance of the methanol/chlorophyll a extract was read at a wavelength 663 nm. The concentration was determined by the following equation: C (μg/mL) = A × 12.7, where C is the chlorophyll concentration, A is the absorbance read and 12.7 is the absorbance coefficient for chlorophyll extracted with methanol.

#### 3.2.2. Biomass Productivity (Dry Biomass)

The biomass was determined in triplicate (2 mL) samples which were washed with ultra-pure water and centrifuged at 4000× *g* for 10 min. The pellet was suspended in a minimum amount of ultra-pure water (100 μL), then transferred to a previously tared and dried glass to a constant weight at 80 °C overnight. The dry weight of the cells was measured using a precision balance [47].

### 3.3. Optimization of Culture Conditions

As a way of evaluating the best conditions for light intensity and NaNO_3_ concentration in cyanobacteria cultivation, a complete 2^2^ factorial design in triplicate was proposed, defining as independent variables the luminous intensity (X) and the concentration of NaNO_3_ (Y) (Table 4) present in the media cultivation of each strain. 

The response variables were the biomass yield and the lipid content obtained at the end of the cultivation stage, calculated from the following equations:

• Biomass productivity (PDBM):

(mg/L/day) P = (X_t_ − X_0_)/(t − t_0_) (1)

where X_t_ is the dry biomass (g·L^−1^) at time t (day) and X_0_ is the dry biomass (g·L^−1^) at time t_0_ (day)

• Lipid content (%) (TL) [48]:

(2) $$\%\;\mathrm{Lipids}\;=\;\frac{\mathrm{VCl}}{B}\;\times \;100$$

where V (mL) is the volume of chloroform extracted, Cl is the measured lipid concentration (mg/mL) and B (mg/L/day) is the dry biomass used in the extraction.

• Lipid Productivity (PDLP) (mg/L/day) [48]:

PDLP = PDBM × TL/100.(3)

Statistical analysis of the results was performed using the MINITAB program (version 7.0, Minitab, Inc, Philadelphia, PA, United States), considering biomass yield and the lipid content obtained at the end of the growing stage as response variables. The results were expressed in Pareto charts, based on the analysis of the significance of the effects of the independent variables, including the interaction between the variables. The bars are the standardized (*t*-calculated) effects related to biomass yield and lipid content, with a 95% confidence level. The in-response surface graphs are used to search for maximum values of biomass yield and lipid content.

### 3.4. Total Lipids Extraction and Fatty Acid Profile by Gas Chromatography (GC) Analysis

For total lipid extraction, 600 mL of culture after 13 days was centrifuged and lyophilized to obtain the dry biomass. To determine the dry biomass, cyanobacterial suspensions were centrifuged (5500× *g* for 30 min) and lyophilized at −40 °C for 48 h. The point of harvest was late post-exponential. Lipid extraction was performed per Bligh and Dyer [49], with modifications suggested by Chatsungnoen and Chisti [48]. First, a 5.7:3:1 (*v*/*v*) CHCl_3_:MeOH:H_2_O (chloroform/methanol/water) solution was added to the lyophilized biomass, followed by mixing using a mixer until homogeneous, with an extraction time of 2 h. The organic phase located in the bottom was recovered.

Esterification was performed using the AOCS Official Method Ce 2-66to destroy epoxy, hydroperoxy, cyclopropenyl, cyclopropyl, and possibly hydroxyl, and acetylenic fatty acids for the preparation of fatty acid methyl esters. Analysis of the fatty acid composition was performed using a Thermo Scientific Trace 1300 gas chromatograph (GC) (Waltham, MA, USA) coupled to a Thermo Scientific MS-ISQ single quadrupole mass spectrometer (Waltham, MA, USA), with an AI 1310 autosampler equipped with a capillary column ZB-5HT (30 m × 0.25 mm × 0.1 μm). Helium gas was used as the carrier, at a flow rate of 1 mL/min. The sample injection was 1.0 μL in splitless mode.

The injector was operated at 220 °C and the oven temperature started at 40 °C, raising to 200 °C (8 °C/min), then holding for 1 min, then rising to 300 °C (15 °C/min), holding for 5 min, then rising again to 350 °C (15 °C/min), and finally holding for another 9 minutes. The MS-ISQ operated with the following settings: Transfer line at 280 °C, ionization source at 280 °C, mass band (40–1,000 Da) with a 1 scan/s scan, and electronic ionization at 70 eV.

The identification of fatty acids was carried out by comparing the mass spectra with those of the commercial libraries NIST2011, WILEY2009, and FAMES2011. Lipid concentration was calculated by calculating the peak area normalization and validation of retention indices by calculating retention indices from homologous hydrocarbons.

### 3.5. Biodiesel Properties Based on the Fatty Acid Profile

The biodiesel properties were estimated using “BiodieselAnalyzer Ver. 2.2 (available at http://www.brteam.ir/biodieselanalyzer).

## 4. Conclusions

This study documented the experimental design using two independent variables, NaNO_3_ concentration (1 to 2 g/L) and luminous intensity (15 to 100 µmol photons m^−2^ s^−1^), which allowed us to optimize the culture conditions based on the dependent variables of biomass productivity and lipid content. For all strains, the variation of both independent variables was statistically significant. For the lipid content, the NaNO_3_ concentration for CACIAM05 and the level of both independent variables for CACIAM18 was not statistically significant.

The studied strains showed higher biomass productivity at high levels of both variables (NaNO_3_ concentration at 2 g/L and luminous intensity equivalent to 100 µmol photons m^−2^ s^−1^). For the lipid content, the CACIAM05, CACIAM18, and CACIAM25 reached optimal conditions at a high level of light intensity (100 µmol photons m^−2^ s^−1^) and a low level of NaNO_3_ concentration (1 g/L), and CACIAM08 at high levels of both variables (NaNO_3_ concentration at 2 g/L and luminous intensity equivalent to 100 µmol photons m^−2^ s^−1^).

Regarding the chemical composition of fatty acids, it was possible to identify the optimal cultivation conditions to obtain a better-quality biodiesel at low levels of both variables (NaNO_3_ concentration at 1 g/L and luminous intensity equivalent to 15 µmol photons m^−2^ s^−1^) and for CACIAM08 and CACIAM18 at a high level of light intensity (100 µmol photons m^−2^ s^−1^) and low NaNO_3_ concentration (1 g/L).

Based on the results obtained, the potential for the use of cyanobacteria lipids with optimal cultivation conditions as a promising source of biodiesel production was confirmed. The next stage of studies will consider scaling up production to produce sufficient oil for biodiesel synthesis, to experimentally determine the biodiesel physicochemical parameters and to compare the results with those obtained with software.

## Figures and Tables

**Table 1:** Experimental matrix and results obtained from the experimental design (2^2^) used to optimize the independent variables, light intensity (X), and concentration of NaNO_3_ (Y) in the culture medium.

**Assay**	**Variable**				**Biomass Productivity (mg/L/day)**				**Lipid Content (%)**				**Lipid Productivity (mg/L/day)**			
					**CACIAM ^2^**				**CACIAM**				**CACIAM**			
	**Coded**		**Real ^1^**		05	08	18	25	05	08	18	25	05	08	18	25
	**X**	**Y**	**X**	**Y**												
1	−	−	15	1	26.8	12.1	23.2	24.2	15.3	12.4	20.6	33.1	4.1	3.8	4.8	3.4
2	−	+	15	2	37.4	15.3	33.2	42.1	18.9	31.7	32.9	14.4	7.1	2.3	10.9	24.6
3	+	−	100	1	39.5	16.3	34.7	39.0	**25.6**	15.0	**37.9**	**58.3**	10.1	7.2	13.2	11.1
4	+	+	100	2	48.4	18.4	45.8	53.7	17.9	**44.0**	20.7	28.4	8.7	3.3	9.5	7.4
5	0	0	57.5	1.5	34.6	9.5	34.2	30.5	18.1	18.1	13.7	7.8	6.3	1.7	4.7	2.4
6	0	0	57.5	1.5	36.1	10.2	34.0	29.7	18.7	18.9	14.9	8.5	6.7	1.9	5.1	2.5
7	0	0	57.5	1.5	35.8	9.2	34.6	31.6	17.6	17.3	12.4	7.0	6.3	1.6	4.3	2.2

^1^ X = Luminous intensity (µmol photons m^−2^ s^−1^) and Y = NaNO_3_ concentration (g/L). ^2^ *Synechocystis* sp. CACIAM05, *Microcystis aeruginosa* CACIAM08, *Pantanalinema rosaneae* CACIAM18, and *Limnothrix* sp. CACIAM25.

**Table 2:** Lipidic fatty acid profile (%) extracted from cyanobacteria strains.

Strains	Fatty Acid												
	C12:0	C14:0	C16:0	C16:1	C17:0	C18:0	C18:1	C18:2	C18:3	C20:0	SFA	MUFA	PUFA
*Synechocystis* sp. CACIAM05 NaNO_3_ +/Light +	9.16	7.08	54.96	-	-	9.62	-	-	-	-	**80.82**	0.00	0.00
*Synechocystis* sp. CACIAM05 NaNO_3_ +/Light −	-	-	29.90	2.13	2.13	-	42.65	-	-	-	32.03	44.78	0.00
*Synechocystis* sp. CACIAM05 NaNO_3_ −/Light +	-	-	37.65	2.25	2.27	-	28.15	-	-	-	39.92	30.41	0.00
*Synechocystis* sp. CACIAM05 NaNO_3_ −/Light −	-	-	37.77	2.01	2.06	-	20.55	-	-	-	39.83	22.56	0.00
*Synechocystis* sp. CACIAM05 NaNO_3_ 0/Light 0	-	-	60.52	-	-	2.62	-	14.65	2.96	-	63.14	0.00	17.61
*M. aeruginosa* CACIAM08 NaNO_3_ +/Light +	-	-	29.97	-	-	15.32	49.50	-	-	1.33	46.62	49.50	0.00
*M. aeruginosa* CACIAM08 NaNO_3_ +/Light −	-	-	25.71	-	28.36	-	43.90	-	-	-	**54.07**	**43.90**	0.00
*M. aeruginosa* CACIAM08 NaNO_3_ −/Light +	-	-	-	-	23.77	5.69	5.81	-	-	-	29.47	5.81	0.00
*M. aeruginosa* CACIAM08 NaNO_3_ −/Light −	-	1.62	33.14	-	11.25	11.20	15.42	-	-	1.39	58.61	15.42	0.00
*M. aeruginosa* CACIAM08 NaNO_3_ 0/Light 0	-	-	31.18	-	20.67	3.95	29.87	-	-	-	55.80	29.87	0.00
*Pantanalinema rosaneae* CACIAM18 NaNO_3_ +/Light +	-	9.92	32.99	34.13	-	-	4.59	-	-	-	42.92	38.72	0.00
*Pantanalinema rosaneae* CACIAM18 NaNO_3_ +/Light −	-	9.91	25.22	52.55	-	-	-	-	-	-	35.13	52.55	0.00
*Pantanalinema rosanea* CACIAM18 NaNO_3_ −/Light +	-	18.58	29.66	23.79	-	-	-	-	-	-	48.23	23.79	0.00
*Pantanalinema rosaneae* CACIAM18 NaNO_3_ −/Light −	-	14.91	32.74	42.96	-	2.42	-	-	-	-	**50.06**	**42.957**	0.00
*Pantanalinema rosaneae* CACIAM18 NaNO_3_ 0/Light 0	-	18.10	29.86	25.51	-	-	7.79	-	-	-	47.97	33.30	0.00
*Limnothrix* sp. CACIAM25 NaNO_3_ +/Light +	-	3.59	22.12	5.11	20.54	9.44	33.33	-	-	1.15	56.83	38.44	0.00
*Limnothrix* sp. CACIAM25 NaNO_3_ +/Light −	-	-	39.83	-	12.72	8.97	38.49	-	-	-	**61.51**	**38.49**	0.00
*Limnothrix* sp. CACIAM25 NaNO_3_ −/Light +	-	10.62	43.29	20.93	-	3.47	-	13.05	-	-	57.38	20.93	13.05
*Limnothrix* sp. CACIAM25 NaNO_3_ −/Light −	-	1.62	33.14	-	11.25	11.20	15.42	-	-	1.39	48.15	33.08	0.00
*Limnothrix* sp. CACIAM25 NaNO_3_ 0/Light 0	-	-	23.39	3.43	29.45	-	32.88	-	-	-	52.84	36.31	0.00

+ = high level, - = low level, 0 = central level for the variables, referring to NaNO_3_ concentration and light intensity. SFA: Saturated fatty acids, MUFA: Monounsaturated fatty acids, PUFA: Polyunsaturated fatty acids.

**Table 3:** Empirical parameters of biodiesel quality based on the fatty acid profile and specifications based on Brazilian, European and United States (US) standards.

Strains	DU	SV (mg/g)	IV	CN	LCSF	CFPP (°C)	CP (°C)	PP (°C)	APE	BAPE	HHV	ν (mm²/s)	ρ (g/cm³)
CACIAM05 NaNO_3_ +/Light +	0.0	181.9	0.0	76.3	10.3	15.9	23.9	19.1	0.0	0.0	31.5	2.8	0.7
CACIAM05 NaNO_3_ +/Light −	44.8	159.0	40.5	71.5	3.0	−7.1	10.7	4.8	42.7	0.0	30.3	2.9	0.7
CACIAM05 NaNO_3_ −/Light +	30.4	147.7	27.6	77.1	3.8	−4.7	14.8	9.3	28.2	0.0	27.7	2.6	0.6
CACIAM05 NaNO_3_ −/Light −	22.6	131.9	20.5	83.1	3.8	−4.6	14.9	9.3	20.6	0.0	24.5	2.3	0.5
CACIAM05 NaNO_3_ 0/Light 0	35.2	172.5	34.6	70.1	7.4	6.7	26.8	22.3	35.2	20.6	31.7	2.9	0.7
CACIAM08 NaNO_3_ +/Light +	49.5	196.2	44.5	64.1	12.0	21.2	10.8	4.9	49.5	2.7	38.0	3.9	0.8
CACIAM08 NaNO_3_ +/Light −	43.9	201.9	39.5	64.5	2.6	−8.4	8.5	2.4	43.9	0.0	38.7	4.0	0.9
CACIAM08 NaNO_3_ −/Light +	5.8	72.0	5.2	121.0	2.9	−7.5	−5.0	−12.2	5.8	0.0	14.0	1.7	0.3
CACIAM08 NaNO_3_ −/Light −	15.4	154.9	13.9	78.4	10.3	15.9	12.4	6.7	15.4	2.8	29.2	2.9	0.6
CACIAM08 NaNO_3_ 0/Light 0	29.9	177.9	26.9	70.9	5.1	−0.5	11.4	5.6	29.9	0.0	33.8	3.3	0.7
CACIAM18 NaNO_3_ +/Light +	38.7	180.6	38.2	67.9	3.3	−6.1	12.4	6.6	4.6	0.0	31.8	2.7	0.7
CACIAM18 NaNO_3_ +/Light −	52.6	195.0	52.5	62.5	2.5	−8.6	8.3	2.2	0.0	0.0	34.1	2.8	0.8
CACIAM18 NaNO_3_ −/Light +	23.8	162.7	23.8	74.5	3.0	−7.2	10.6	4.7	0.0	0.0	28.0	2.4	0.6
CACIAM18 NaNO_3_ −/Light −	43.0	207.4	42.9	63.0	4.5	−2.4	12.2	6.5	0.0	0.0	36.2	3.1	0.8
CACIAM18 NaNO_3_ 0/Light 0	33.3	181.2	32.5	69.1	3.0	−7.1	10.7	4.8	7.8	0.0	31.7	2.7	0.7
CACIAM25 NaNO_3_ +/Light +	38.4	197.7	35.1	66.0	8.1	8.9	6.6	0.4	33.3	2.3	37.6	3.8	0.8
CACIAM25 NaNO_3_ +/Light −	38.5	207.3	34.6	64.9	8.5	10.1	16.0	10.5	38.5	0.0	39.5	4.1	0.9
CACIAM25 NaNO_3_ −/Light +	47.0	199.5	44.5	63.6	6.1	2.6	17.8	12.5	26.1	13.1	35.7	3.1	0.8
CACIAM25 NaNO_3_ −/Light −	33.1	167.7	30.1	72.1	6.3	3.4	6.8	0.5	30.0	0.0	32.1	3.2	0.7
CACIAM25 NaNO_3_ 0/Light 0	36.3	184.8	33.0	68.4	2.3	−9.1	7.3	1.1	32.9	0.0	35.2	3.5	0.8
**Biodiesel Quality Specifications**													
ABNT NBR			Report	Report		Country specific	-	-				3.0 (min.)/6.0 (max.)	-
EU EN 14214			120 (max.)	51 (min.)		Country specific	-	Country specific				3.5 (min.)/5.0 (max.)	0.86 (min.)/0.90 (max.)
USA ASTM D6751			-	47 (min.)		-	Report	-				1.9 (min.)/6.0 (max.)	-

DU: degree of unsaturation, SV: saponification value, IV: iodine value, CN: cetane number, LCSF: long chain saturated factor, CFPP: cold filter plugging point, CP: cloud point, PP: pour point, APE: allylic position equivalent, BAPE: bis-allylic position equivalent, HHV: higher heating value, ν: kinematic viscosity, ρ: density.

**Table 4:** Real and coded levels for the variables: luminous intensity and nitrogen levels (NaNO_3_) in the cultivation of cyanobacteria.

Variable		Level		
**Independent Variables**	Symbol	-	0	+
**Light Intensity (µmol photons m^−2^ s^−1^)**	X	15	57.5	100
**NaNO_3_ (g/L)**	Y	1.0	1.5	2.0

## Supplementary Materials

The following are available online at https://www.mdpi.com/1420-3049/24/12/2326/s1. Table S1: Analysis of variance (ANOVA) for the biomass productivity response variable (mg/L/day) of *Synechocystis* sp. CACIAM05, at the 95% confidence level. Table S2: Analysis of variance (ANOVA) for the biomass productivity response variable (mg/L/day) of *Microcystis aeruginosa* CACIAM08, at the 95% confidence level. Table S3: Analysis of variance (ANOVA) for the biomass productivity response variable (mg/L/day) of *Pantanalinema rosaneae* CACIAM18, at the 95% confidence level. Table S4: Analysis of variance (ANOVA) for the biomass productivity response variable (mg/L/day) of *Limnothrix* sp. CACIAM25, at the 95% confidence level. Table S5: Analysis of variance (ANOVA) for the lipid content response variable (%) of *Synechocystis* sp. CACIAM05, at the 95% confidence level. Table S6: Analysis of variance (ANOVA) for the lipid content response variable (%) of *Microcystis aeruginosa* CACIAM08, at the 95% confidence level. Table S7: Analysis of variance (ANOVA) for the lipid content response variable (%) of *Pantanalinema rosaneae* CACIAM18, at the 95% confidence level. Table S8: Analysis of variance (ANOVA) for the lipid content response variable (%) of *Limnothrix* sp. CACIAM25, at the 95% confidence level.
