# Supplementary material for: Optimization of Light Intensity and NaNO3 Concentration in Amazon Cyanobacteria Cultivation to Produce Biodiesel
Source: Molecules. 2019 Jun 24;24(12):2326. doi: 10.3390/molecules24122326 (PMC6630786; doi:10.3390/molecules24122326)
Supplement: Supplementary file 1 [file molecules-24-02326-s001.zip › molecules-492574-sup.pdf]

Table S1 Analysis of variance (ANOVA) for the biomass productivity response variable (mg/L/day) of *Synechocystis* sp. CACIAM05, at the 95% confidence level.

Variable	DF	Adj SS	Adj MS	F-Value	P-Value
**Model**	4	246.492	61.623	103.11	**0.010**
X_1	1	140.186	140.186	234.57	0.004
X_2	1	94.868	94.868	158.74	0.006
X_1X_2	1	0.624	0.624	1.04	0.414
**Curvature**	1	10.815	10.815	18.10	**0.051**
Error	2	1.195	0.598		
Total	6	247.687			
**R^2**	**0.9952**				

X_1= light intensity, **X_2**= nitrogen source, **F**= comparative statistical test in the assays to evaluate the quality of fit of the model.

Table S2 Analysis of variance (ANOVA) for the biomass productivity response variable (mg/L/day) of *Microcystis aeruginosa* CACIAM08, at the 95% confidence level.

Variable	DF	Adj SS	Adj MS	F-Value	P-Value
**Model**	4	80.6391	20.1598	88.52	**0.011**
X_1	1	13.5792	13.5792	59.63	0.016
X_2	1	6.8906	6.8906	30.26	0.031
X_1X_2	1	0.2756	0.2756	1.21	0.386
**Curvature**	1	59.8936	59.8936	263.00	**0.004**
Error	2	0.4555	0.2277		
Total	6	81.0946			

X_1= light intensity, **X_2**= nitrogen source, **F**= comparative statistical test in the assays to evaluate the quality of fit of the model.

Table S3 Analysis of variance (ANOVA) for the biomass productivity response variable (mg/L/day) of *Leptolyngbya* sp. CACIAM18, at the 95% confidence level.

Variable	DF	Adj SS	Adj MS	F-Value	P-Value
**Model**	4	257.585	64.396	661.83	**0.002**
X_1	1	146.531	146.531	1505.97	0.001
X_2	1	110.776	110.776	1138.50	0.001
X_1X_2	1	0.276	0.276	2.83	0.234
**Curvature**	1	0.002	0.002	0.02	**0.889**
Error	2	0.195	0.097		
Total	6	257.779			
**R^2**	**0.9992**				

X_1= light intensity, **X_2**= nitrogen source, **F**= comparative statistical test in the assays to evaluate the quality of fit of the model.

Table S4 Analysis of variance (ANOVA) for the biomass productivity response variable (mg/L/day) of *Limnothrix redekei* CACIAM25, at the 95% confidence level.

Variable	DF	Adj SS	Adj MS	F-Value	P-Value
**Model**	4	584.565	146.141	161.07	**0.006**
X_1	1	173.054	173.054	190.74	0.005
X_2	1	266.179	266.179	293.38	0.003
X_1X_2	1	2.512	2.512	2.77	0.238
**Curvature**	1	142.819	142.819	157.41	**0.006**
Error	2	1.815	0.907		
Total	6	586.379			

X_1= light intensity, **X_2**= nitrogen source, **F**= comparative statistical test in the assays to evaluate the quality of fit of the model.

Table S5 Analysis of variance (ANOVA) for the lipid content response variable (%) of *Synechocystis* sp. CACIAM05, at the 95% confidence level.

Variable	DF	Adj SS	Adj MS	F-Value	P-Value
**Model**	4	61,0391	15,2598	49,38	**0,020**
X_1	1	21,5296	21,5296	69,67	0,014
X_2	1	4,0401	4,0401	13,07	0,069
X_1X_2	1	32,4900	32,4900	105,13	0,009
**Curvature**	1	2,9794	2,9794	9,64	**0,090**
Error	2	0,6181	0,3090		
Total	6	61,6572			
**R^2**	**0,9900**				

X_1= light intensity, **X_2**= nitrogen source, **F**= comparative statistical test in the assays to evaluate the quality of fit of the model.

Table S6 Analysis of variance (ANOVA) for the lipid content response variable (%) of *Microcystis aeruginosa* CACIAM08, at the 95% confidence level.

Variable	DF	Adj SS	Adj MS	F-Value	P-Value
**Model**	4	761,661	190,415	305,09	**0,003**
X_1	1	55,577	55,577	89,05	0,011
X_2	1	582,981	582,981	934,06	0,001
X_1X_2	1	23,281	23,281	37,30	0,026
**Curvature**	1	99,822	99,822	159,94	**0,006**
Error	2	1,248	0,624		
Total	6	762,909			

X_1= light intensity, **X_2**= nitrogen source, **F**= comparative statistical test in the assays to evaluate the quality of fit of the model.

Table S7 Analysis of variance (ANOVA) for the lipid content response variable (%) of *Leptolyngbya* sp. CACIAM18, at the 95% confidence level.

Variable	DF	Adj SS	Adj MS	F-Value	P-Value
**Model**	4	581,793	145,448	93,78	**0,011**
X_1	1	6,708	6,708	4,32	0,173
X_2	1	6,150	6,150	3,97	0,185
X_1X_2	1	217,562	217,562	140,27	0,007
**Curvature**	1	351,372	351,372	226,54	**0,004**
Error	2	3,102	1,551		
Total	6	584,895			

X_1= light intensity, **X_2**= nitrogen source, **F**= comparative statistical test in the assays to evaluate the quality of fit of the model.

Table S8 Analysis of variance (ANOVA) for the lipid content response variable (%) of *Limnothrix redekei* CACIAM25, at the 95% confidence level.

Variable	DF	Adj SS	Adj MS	F-Value	P-Value
**Model**	4	2146,10	536,53	916,15	**0,001**
X_1	1	384,94	384,94	657,31	0,002
X_2	1	591,95	591,95	1010,78	0,001
X_1X_2	1	30,91	30,91	52,79	0,018
**Curvature**	1	1138,30	1138,30	1943,70	**0,001**
Error	2	1,17	0,59		
Total	6	2147,28			

X_1= light intensity, **X_2**= nitrogen source, **F**= comparative statistical test in the assays to evaluate the quality of fit of the model.
