# Supplementary material for: Optimization of Light Intensity and NaNO3 Concentration in Amazon Cyanobacteria Cultivation to Produce Biodiesel
Source: Molecules. 2019 Jun 24;24(12):2326. doi: 10.3390/molecules24122326 (PMC6630786; doi:10.3390/molecules24122326)
Supplement: Supplementary file 1 [file molecules-24-02326-s001.zip › molecules-492574-supp.pdf]

Table 1 Experimental matrix and results obtained from the experimental design 2^2 used to optimize the independent variables light intensity (X) and concentration of $NaNO_3$ (Y) in the culture medium.

Assay	Variable				Biomass productivity (mg/L/day)				Lipid content (%)				Lipid productivity (mg/L/day)			
					CACIAM[2]				CACIAM				CACIAM			
	Coded		Real[1]		05	08	18	25	05	08	18	25	05	08	18	25
	X	Y	X	Y												
1	-	-	15	1	26.8	12.1	23.2	24.2	15.3	12.4	20.6	33.1	4.1	3.8	4.8	3.4
2	-	+	15	2	37.4	15.3	33.2	42.1	18.9	31.7	32.9	14.4	7.1	2.3	10.9	24.6
3	+	-	100	1	39.5	16.3	34.7	39.0	25.6	15.0	37.9	58.3	10.1	7.2	13.2	11.1
4	+	+	100	2	48.4	18.4	45.8	53.7	17.9	44.0	20.7	28.4	8.7	3.3	9.5	7.4
5	0	0	57.5	1.5	34.6	9.5	34.2	30.5	18.1	18.1	13.7	7.8	6.3	1.7	4.7	2.4
6	0	0	57.5	1.5	36.1	10.2	34.0	29.7	18.7	18.9	14.9	8.5	6.7	1.9	5.1	2.5
7	0	0	57.5	1.5	35.8	9.2	34.6	31.6	17.6	17.3	12.4	7.0	6.3	1.6	4.3	2.2

[1] *X= Luminous intensity (µmol/m^2s) and Y= $NaNO_3$ concentration (g/L)*

[2] *Synechocystis* sp. CACIAM05, *Microcystis aeruginosa* CACIAM08, *Leptolyngbya* sp. CACIAM18 and *Limnothrix redekei* CACIAM25

Table 21 Lipidic Fatty acid profile (%) extracted from cyanobacteria strains

Strains	Fatty acid												
	C12:0	C14:0	C16:0	C16:1	C17:0	C18:0	C18:1	C18:2	C18:3	C20:0	SFA	MUFA	PUFA
Synechocystis sp. CACIAM05 $NaNO_3$ + / Light +	9.16	7.08	54.96	-	-	9.62	-	-	-	-	80.82	0.00	0.00
Synechocystis sp. CACIAM05 $NaNO_3$ + / Light -	-	-	29.90	2.13	2.13	-	42.65	-	-	-	32.03	44.78	0.00
Synechocystis sp. CACIAM05 $NaNO_3$ - / Light +	-	-	37.65	2.25	2.27	-	28.15	-	-	-	39.92	30.41	0.00
Synechocystis sp. CACIAM05 $NaNO_3$ - / Light -	-	-	37.77	2.01	2.06	-	20.55	-	-	-	39.83	22.56	0.00
Synechocystis sp. CACIAM05 $NaNO_3$ 0 / Light 0	-	-	60.52	-	-	2.62	-	14.65	2.96	-	63.14	0.00	17.61
M. aeruginosa CACIAM08 $NaNO_3$ + / Light +	-	-	29.97	-	-	15.32	49.50	-	-	1.33	46.62	49.50	0.00
M. aeruginosa CACIAM08 $NaNO_3$ + / Light -	-	-	25.71	-	28.36	-	43.90	-	-	-	54.07	43.90	0.00
M. aeruginosa CACIAM08 $NaNO_3$ - / Light +	-	-	-	-	23.77	5.69	5.81	-	-	-	29.47	5.81	0.00
M. aeruginosa CACIAM08 $NaNO_3$ - / Light -	-	1.62	33.14	-	11.25	11.20	15.42	-	-	1.39	58.61	15.42	0.00
M. aeruginosa CACIAM08 $NaNO_3$ 0 / Light 0	-	-	31.18	-	20.67	3.95	29.87	-	-	-	55.80	29.87	0.00
Leptolyngbya sp. CACIAM18 $NaNO_3$ + / Light +	-	9.92	32.99	34.13	-	-	4.59	-	-	-	42.92	38.72	0.00
Leptolyngbya sp. CACIAM18 $NaNO_3$ + / Light -	-	9.91	25.22	52.55	-	-	-	-	-	-	35.13	52.55	0.00
Leptolyngbya sp. CACIAM18 $NaNO_3$ - / Light +	-	18.58	29.66	23.79	-	-	-	-	-	-	48.23	23.79	0.00
Leptolyngbya sp. CACIAM18 $NaNO_3$ - / Light -	-	14.91	32.74	42.96	-	2.42	-	-	-	-	50.06	42.957	0.00
Leptolyngbya sp. CACIAM18 $NaNO_3$ 0 / Light 0	-	18.10	29.86	25.51	-	-	7.79	-	-	-	47.97	33.30	0.00
Limnothrix redekei CACIAM25 $NaNO_3$ + / Light +	-	3.59	22.12	5.11	20.54	9.44	33.33	-	-	1.15	56.83	38.44	0.00
Limnothrix redekei CACIAM25 $NaNO_3$ + / Light -	-	-	39.83	-	12.72	8.97	38.49	-	-	-	61.51	38.49	0.00
Limnothrix redekei CACIAM25 $NaNO_3$ - / Light +	-	10.62	43.29	20.93	-	3.47	-	13.05	-	-	57.38	20.93	13.05
Limnothrix redekei CACIAM25 $NaNO_3$ - / Light -	-	1.62	33.14	-	11.25	11.20	15.42	-	-	1.39	48.15	33.08	0.00
Limnothrix redekei CACIAM25 $NaNO_3$ 0 / Light 0	-	-	23.39	3.43	29.45	-	32.88	-	-	-	52.84	36.31	0.00

+= high level, **-**= low level, 0= central level for the variables: $NaNO_3$ concentration and light intensity
SFA: saturated fatty acids, MUFA: monounsaturated fatty acids, PUFA: polyunsaturated fatty acids

Table 3 Empirical parameters of biodiesel quality based on the fatty acid profile of cyanobacterial strains

Strains	DU	SV (mg/g)	IV	CN	LCSF	CFPP (ºC)	CP (ºC)	PP (ºC)	APE	BAPE	HHV	υ (mm²/s)	P (g/cm³)
[1]CACIAM05 $NaNO_3$ + / Light +	0.0	181.9	0.0	76.3	10.3	15.9	23.9	19.1	0.0	0.0	31.5	2.8	0.7
CACIAM05 $NaNO_3$ + / Light -	44.8	159.0	40.5	71.5	3.0	-7.1	10.7	4.8	42.7	0.0	30.3	2.9	0.7
CACIAM05 $NaNO_3$ - / Light +	30.4	147.7	27.6	77.1	3.8	-4.7	14.8	9.3	28.2	0.0	27.7	2.6	0.6
CACIAM05 $NaNO_3$ - / Light -	22.6	131.9	20.5	83.1	3.8	-4.6	14.9	9.3	20.6	0.0	24.5	2.3	0.5
CACIAM05 $NaNO_3$ 0 / Light 0	35.2	172.5	34.6	70.1	7.4	6.7	26.8	22.3	35.2	20.6	31.7	2.9	0.7
[2]CACIAM08 $NaNO_3$ + / Light +	49.5	196.2	44.5	64.1	12.0	21.2	10.8	4.9	49.5	2.7	38.0	3.9	0.8
CACIAM08 $NaNO_3$ + / Light -	43.9	201.9	39.5	64.5	2.6	-8.4	8.5	2.4	43.9	0.0	38.7	4.0	0.9
CACIAM08 $NaNO_3$ - / Light +	5.8	72.0	5.2	121.0	2.9	-7.5	-5.0	-12.2	5.8	0.0	14.0	1.7	0.3
CACIAM08 $NaNO_3$ - / Light -	15.4	154.9	13.9	78.4	10.3	15.9	12.4	6.7	15.4	2.8	29.2	2.9	0.6
CACIAM08 $NaNO_3$ 0 / Light 0	29.9	177.9	26.9	70.9	5.1	-0.5	11.4	5.6	29.9	0.0	33.8	3.3	0.7
[3]CACIAM18 $NaNO_3$ + / Light +	38.7	180.6	38.2	67.9	3.3	-6.1	12.4	6.6	4.6	0.0	31.8	2.7	0.7
CACIAM18 $NaNO_3$ + / Light -	52.6	195.0	52.5	62.5	2.5	-8.6	8.3	2.2	0.0	0.0	34.1	2.8	0.8
CACIAM18 $NaNO_3$ - / Light +	23.8	162.7	23.8	74.5	3.0	-7.2	10.6	4.7	0.0	0.0	28.0	2.4	0.6
CACIAM18 $NaNO_3$ - / Light -	43.0	207.4	42.9	63.0	4.5	-2.4	12.2	6.5	0.0	0.0	36.2	3.1	0.8
CACIAM18 $NaNO_3$ 0 / Light 0	33.3	181.2	32.5	69.1	3.0	-7.1	10.7	4.8	7.8	0.0	31.7	2.7	0.7
[4]CACIAM25 $NaNO_3$ + / Light +	38.4	197.7	35.1	66.0	8.1	8.9	6.6	0.4	33.3	2.3	37.6	3.8	0.8
CACIAM25 $NaNO_3$ + / Light -	38.5	207.3	34.6	64.9	8.5	10.1	16.0	10.5	38.5	0.0	39.5	4.1	0.9
CACIAM25 $NaNO_3$ - / Light +	47.0	199.5	44.5	63.6	6.1	2.6	17.8	12.5	26.1	13.1	35.7	3.1	0.8
CACIAM25 $NaNO_3$ - / Light -	33.1	167.7	30.1	72.1	6.3	3.4	6.8	0.5	30.0	0.0	32.1	3.2	0.7
CACIAM25 NaNO3 0 / Light 0	36.3	184.8	33.0	68.4	2.3	-9.1	7.3	1.1	32.9	0.0	35.2	3.5	0.8

[1]*Synechocystis* sp. CACIAM05, [2]*Microcystis aeruginosa* CACIAM08, [3]*Leptolyngbya* sp. CACIAM18, [4]Limnothrix *redekei* CACIAM25

Table 5 Strain sources used in this study.

Strain	Local
Microcystis aeruginosa CACIAM03	Hydroelectric plant of Tucuruí reservoir
Synechocystis sp. CACIAM05	Bologna Lake
Lyngbya sp. CACIAM07	Hydroelectric plant of Tucuruí reservoir
Microcystis aeruginosa CACIAM08	Hydroelectric plant of Tucuruí reservoir
Synechococcus sp. CACIAM66	Hydroelectric plant of Tucuruí reservoir
Leptolyngbya sp. CACIAM18	Bologna Lake

Limnothrix redekei CACIAM25	Hydroelectric plant of Tucuruí reservoir
Planktothrix pseudoagardhii CACIAM27	Hydroelectric plant of Tucuruí reservoir

Table 6 Real and coded levels for the variables: luminous intensity and nitrogen levels ($NaNO_3$) in the cultivation of cyanobacteria

Variable		Level		
Independent variables	Symbol	-1	0	1
Light intensity (µmol/m^{2}s)	X	15	57.5	100
$NaNO_3$ (g/L)	Y	1	1.5	2.0
